# Carbon Dioxide Monitoring Demonstrates Variations in the Quality of Ventilation on Public Transportation Buses and University Student Shuttle Vans and Identifies Effective Interventions

**DOI:** 10.20411/pai.v8i1.619

**Published:** 2023-11-21

**Authors:** David Henry Greentree, Brigid M. Wilson, Curtis J. Donskey

**Affiliations:** 1 Department of Biology, College of Arts and Sciences, Case Western Reserve University, Cleveland, Ohio; 2 College of Medicine, The Ohio State University, Columbus, Ohio; 3 Geriatric Research, Education and Clinical Center, Louis Stokes Veterans Affairs Medical Center, Cleveland, Ohio; 4 School of Medicine, Case Western Reserve University, Cleveland, Ohio

**Keywords:** COVID-19, SARS-CoV-2, ventilation, carbon dioxide, transportation

## Abstract

**Background:**

There is a risk for transmission of severe acute respiratory syndrome 2 (SARS-CoV-2) and other respiratory viruses in motor vehicles, particularly if ventilation is inadequate.

**Methods:**

We used carbon dioxide monitoring to examine the quality of ventilation in several public transportation buses and in university student shuttle vans in the Cleveland metro area during peak and non-peak travel times. Carbon dioxide levels above 800 parts per million (ppm) were considered an indicator of suboptimal ventilation for the number of people present. In the shuttle vans, we evaluated the impact of an intervention to improve ventilation.

**Results:**

In large articulated buses with 2 ventilation systems, carbon dioxide concentrations never exceeded 800 ppm, whereas in standard buses with 1 ventilation system concentrations rose above 800 ppm during peak travel times and on some trips during non-peak travel times. In shuttle vans, the ventilation system was not turned on during routine operation, and carbon dioxide levels rose above 800 ppm on all trips during peak and non-peak travel times. In the shuttle vans, an intervention involving operation of the existing ventilation system resulted in a significant reduction in carbon dioxide levels (mean concentration, 1,042 no intervention versus 785 with intervention; *P* < 0.001).

**Conclusions:**

Our findings demonstrate substantial variability in the quality of ventilation in public transportation buses and university shuttle vans. There is a need for efforts to assess and optimize ventilation in motor vehicles used for public transportation to reduce the risk for aerosol-mediated transmission of respiratory viruses. Carbon dioxide monitoring may provide a useful tool to assess and improve ventilation.

## INTRODUCTION

Indoor spaces that are inadequately ventilated pose a risk for acquisition of severe acute respiratory syndrome coronavirus 2 (SARS-CoV-2) and other respiratory viruses [1, 2]. It has therefore been recommended that measures be taken to assess and improve ventilation in all commercial buildings and schools [3, 4]. There is some evidence that such interventions may reduce the risk for coronavirus disease 2019 (COVID-19) infections. In a study of elementary schools in Georgia, the incidence of COVID-19 was significantly lower in schools that improved ventilation using methods to dilute (eg, opening windows) and/or filter airborne particles [5]. In a large study in Italy, the risk of COVID-19 was reduced by at least 74% in classrooms equipped with mechanical ventilation systems in comparison to classrooms with only natural ventilation [6].

In addition to buildings, there is a risk for transmission of respiratory viruses in motor vehicles [7–9]. Elevated COVID-19 mortality rates have been reported among transportation workers in comparison to workers in other occupations [10]. Bus drivers have been reported to have a relatively high risk for COVID-19 or severe COVID-19 in comparison to other professions [11, 12]. In buses and patient transport vans, transmission of SARS-CoV-2 from source patients to individuals seated more than 1.8 meters away has been reported, suggesting possible aerosol-mediated transmission [13–15]. In cars, ventilation was poor under several common driving conditions (eg, parked or city driving with windows closed and ventilation system fan off, ventilation system operating on recirculation mode) based on carbon dioxide monitoring [7].

The Centers for Disease Control and Prevention (CDC) and some experts in aerosol science have recommended passive carbon dioxide monitoring as a practical tool to assess the adequacy of ventilation in occupied indoor spaces [2, 3]. Carbon dioxide levels rise in occupied spaces with suboptimal ventilation for the number of people present because the concentration of carbon dioxide in exhaled breath is approximately 40,000 parts per million (ppm) vs 420 ppm in outdoor air [1, 16]. Carbon dioxide monitoring has been used to assess and improve ventilation in areas such as schools, university buildings, dental offices, and residential homes [17–20].

Public transportation buses and vans are an essential service in urban and rural communities. The quality, design, and utilization of heating, ventilation, and air conditioning (HVAC) systems of buses and vans could impact the risk for viral exposure. Here, we assessed the quality of ventilation in several public bus and university shuttle van transport systems in the Cleveland, Ohio, metro area using carbon dioxide monitoring. In the shuttle vans, we evaluated the impact of an intervention to improve ventilation.

## METHODS

### Evaluation of Ventilation on the Public Transportation Buses and Vans

The study was approved as a quality improvement project by the Cleveland VA Medical Center's Research and Development Committee. No identifiable information was collected for individuals using the buses or vans. We used carbon dioxide measurements to assess adequacy of ventilation in 7 public transportation lines during both peak (defined as above 50% of the maximum recommended passenger number for at least 15 minutes) and non-peak (defined as not exceeding 50% of the maximum recommended passenger number for at least 15 minutes) travel times. A minimum of 4 trips were taken during peak travel times and 4 during non-peak travel times for each type of vehicle. The study was conducted between September 2022 and January 2023. A member of the research team carried a handheld IAQ-MAX CO2 monitor and data logger (CO2Meter) that recorded carbon dioxide levels once per minute later rounded to the nearest 10 ppm. The research member rode each of the lines for at least 40 minutes while staying in the nearest available seat to the driver. The number of passengers was recorded every minute. Carbon dioxide readings above 800 ppm were considered an indicator of suboptimal ventilation for the number of people present [1]. The devices were calibrated daily in outdoor air per the manufacturer's recommendations.

**Figure 1.**
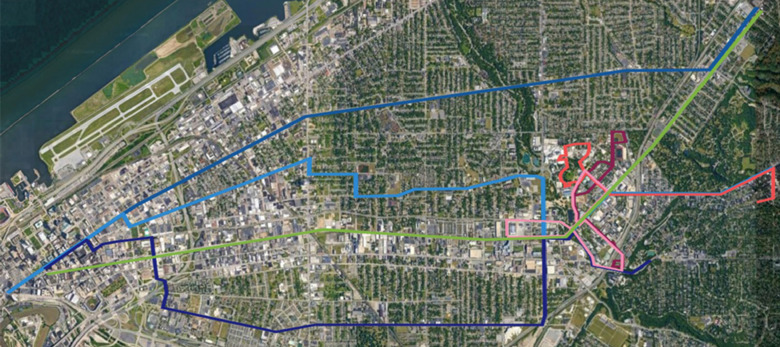
**Map of Cleveland, Ohio, area with highlighted public transportation routes.** The green line shows the articulated bus route. The blue lines show the standard bus routes. The red, orange, and pink lines show the shuttle van routes.

Of the 7 transportation lines tested, 1 was categorized as an articulated bus (ie, a high-passenger capacity bus with a joint in the middle to increase maneuverability), 3 as standard buses, and 3 as shuttle vans. The buses were operated by a local government agency and the shuttle vans were operated by a university. University students were the primary users of the university shuttle vans. The routes for each transportation line are shown in Figure 1. The articulated buses were of an Xcelsior CNG model with a maximum recommended passenger number of 60 and 2 integrated rooftop bus HVAC units. The frontal HVAC unit was an RLF Series Rooftop HVAC System for Coach and Transit (Thermo King) model with an adjustable listed circulation capacity of either 4,078, 2,328, or 1,699 cubic meters per hour, and the back unit was a TE-15 model (Thermo King) with an adjustable listed circulation capacity of either 3,814 or 2,366 cubic meters per hour. The standard buses were Gillig Low-Floor BRT models with a maximum recommended passenger number of 40 and 1 integrated rooftop TE-15 HVAC unit. The shuttle vans had a maximum recommended passenger number of 15 and a single EZ3 HVAC unit (American Cooling Technology, Inc.) with a single listed circulation capacity of 1,186 cubic meters per hour. The HVAC units in the shuttle van were rarely turned on, whereas the articulated and standard buses had their HVAC units continuously running.

In motion, all vehicles proceeded at less than 35 miles per hour according to road restrictions with no obvious speed differences between vehicle categories. All vehicles had their windows closed on all rides. During the study, masks were optional and infrequently worn on the articulated and standard buses, whereas mask wearing was mandatory and strictly enforced on all shuttles.

### Intervention to Improve Ventilation on the Shuttle Vans

Our initial evaluation suggested that the shuttle vans had suboptimal ventilation even for trips during non-peak travel times. During all trips, the EZ3 HVAC units were off, and the windows were closed. Thus, we assessed the impact of an intervention to improve ventilation in the vans. Previous studies have shown that opening windows can markedly improve ventilation in motor vehicles [7]. However, this was not an option as the sliding windows in the vans were bolted shut.

For the intervention, the researcher asked the drivers of the shuttle vans to turn on the existing EZ3 HVAC system to either the medium or high setting and maintain at that level throughout the trip with no other change from routine operations. The concentration of carbon dioxide was monitored during 4 trips with the HVAC system operating. The mean and peak concentrations of carbon dioxide were compared for the 4 intervention trips when the HVAC system was operating vs 4 trips when the HVAC was not turned on. We hypothesized that operation of the HVAC system would prevent elevation of carbon dioxide above 800 ppm during peak ridership hours [7].

### Data Analysis

We considered measures obtained within the same ride to be independent of each other. For each vehicle type, we calculated Pearson's correlation coefficients to assess the correlation between the percentage passenger capacity (number of passengers/maximum recommended number of passengers x 100) and the carbon dioxide levels during peak and non-peak travel times. Unpaired Student's t-test with unequal variance was used to compare concentrations of carbon dioxide during peak vs non-peak travel times. To assess the impact of the intervention, we predicted carbon dioxide levels with percentage passenger capacity before and after the intervention using a linear regression model with an interaction term to test if the intervention altered the association between passenger capacity and carbon dioxide levels or if a shift in carbon dioxide levels was observed without a detected difference in slope. All analyses were performed in R Version 4.3.2

## RESULTS

### Evaluation of Ventilation on Public Transportation Buses and Vans

**Figure 2.**
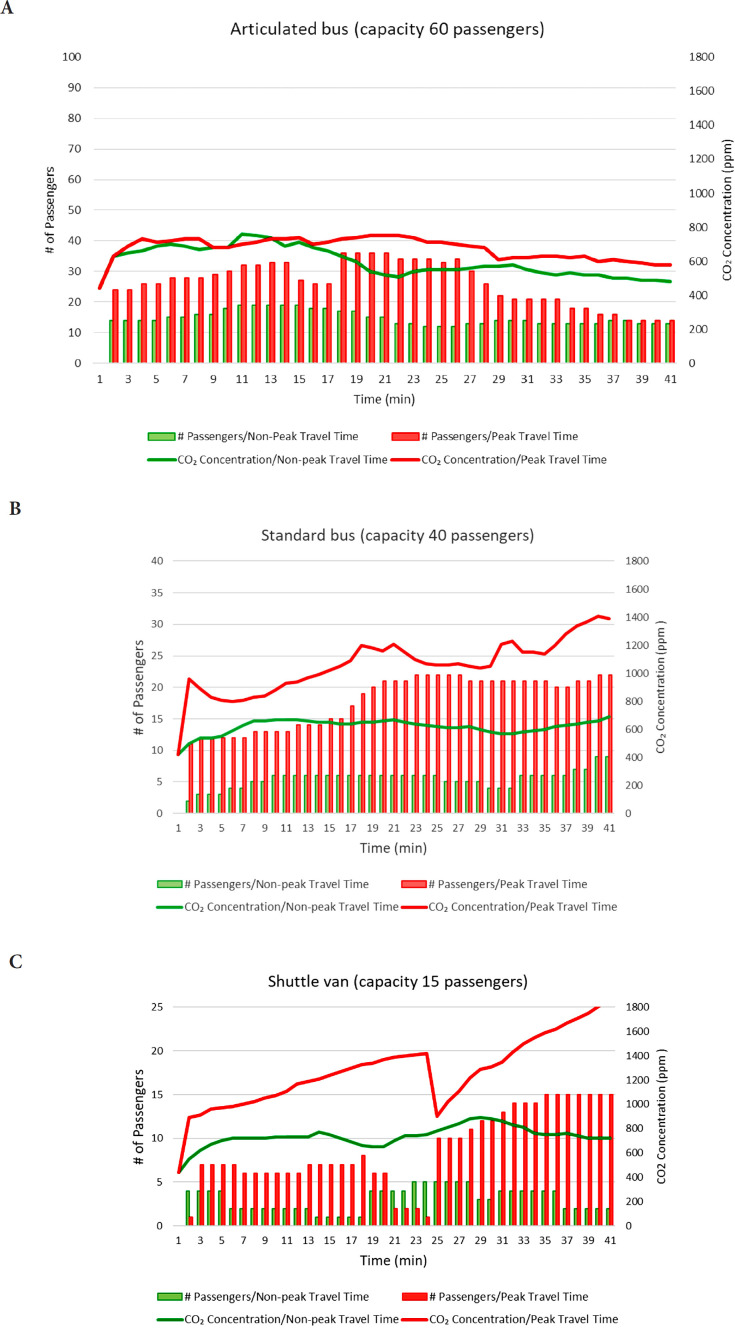
**Carbon dioxide concentrations and passenger numbers over time during 6 individual trips, including 1 during peak travel time and 1 during non-peak travel time for the articulated bus (A), standard bus (B), and shuttle van (C).** Abbreviations: CO_2_, carbon dioxide; ppm, parts per million; min, minutes.

Outdoor carbon dioxide concentrations measured during calibration of the devices were consistently approximately 440 to 450 ppm. Figure 2 shows carbon dioxide concentrations and passenger numbers over time during 6 of the trips, including 1 during peak travel time and 1 during non-peak travel time for each type of vehicle. After the research staff member entered the articulated or standard buses, the concentration of carbon dioxide rose rapidly from ~450 ppm (outdoor air) to a higher level and then remained relatively stable throughout the trip. In the shuttle van, carbon dioxide concentrations remained relatively stable during a non-peak travel time trip, whereas the concentration of carbon dioxide increased steadily to ~1,700 ppm during a peak travel time trip (Figure 2C). The carbon dioxide concentration in the shuttle van did not fluctuate substantially during brief periods when the number of passengers temporarily increased or decreased dramatically. For example, there was a substantial reduction in the number of passengers on the shuttle van during the peak travel time trip between minutes 18 and 24, but the carbon dioxide level only decreased transiently by ~100 ppm from minutes 23 to 27.

**Figure 3.**
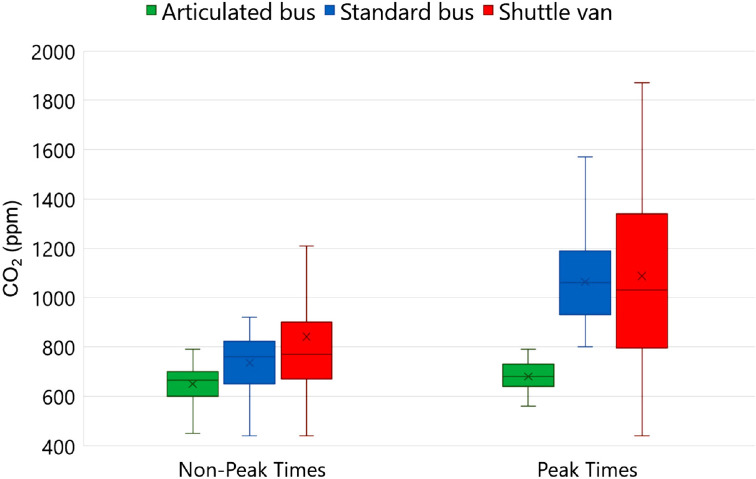
**Comparison of carbon dioxide concentrations during non-peak vs peak travel times in each of the vehicle types.** Solid rectangles indicate second quartile (50% of values). Horizontal lines within rectangles indicate median values and X markers indicate mean values. Whiskers indicate lower and upper quartiles. Abbreviations: CO2, carbon dioxide; ppm, parts per million.

Figure 3 provides a comparison of carbon dioxide concentrations during non-peak vs peak travel times in each of the vehicle types. Carbon dioxide concentrations were significantly higher during peak vs non-peak travel times in the standard buses and shuttle vans (*P*<0.001). In the articulated buses (8 trips), the carbon dioxide concentrations always remained below 800 ppm during peak (peak carbon dioxide concentration, 760 ppm) and non-peak (peak carbon dioxide concentration, 790 ppm) travel times (*P*>0.05). In the standard buses, suboptimal ventilation (peak carbon dioxide concentration above 800 ppm) occurred during all 5 trips during peak travel times (peak carbon dioxide concentration, 1,600 ppm) and during 2 of 4 (50%) trips during non-peak travel times (peak carbon dioxide concentration, 920 ppm). On the shuttle vans, all 8 trips had suboptimal ventilation, including 4 during peak travel times (peak carbon dioxide concentration, 1,870 ppm) and 4 during non-peak travel times (peak carbon dioxide concentration, 1,360 ppm). Even on trips where the research member was the only shuttle van passenger, carbon dioxide levels reached as high as 1,420 ppm.

**Figure 4.**
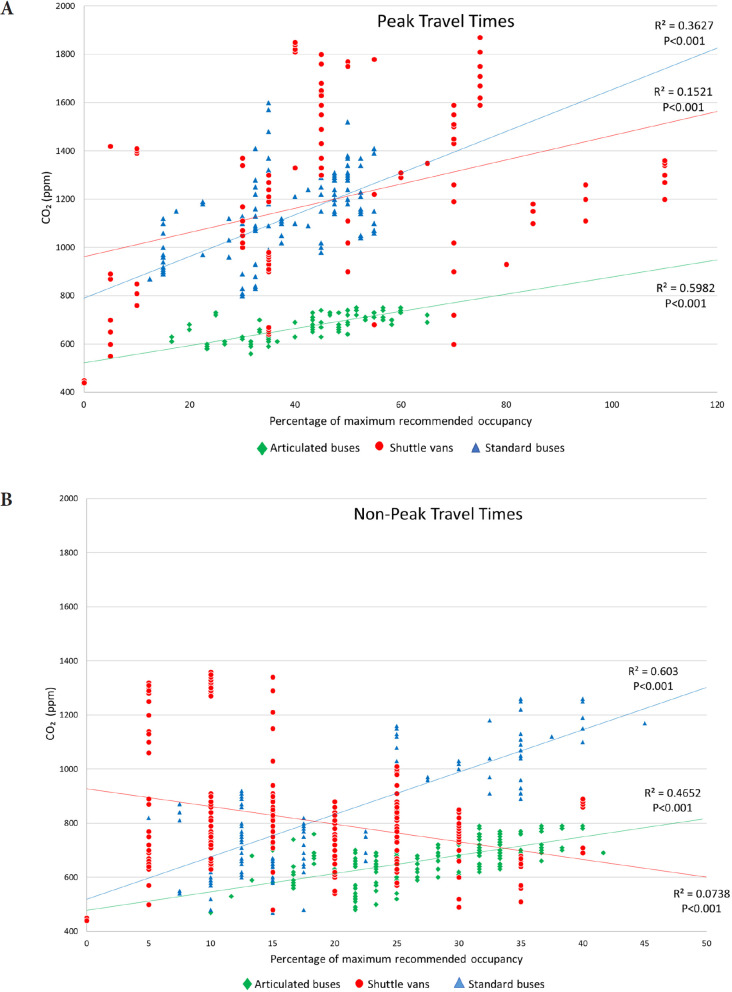
**Correlation between the concentration of carbon dioxide and the percentage of maximum recommended passenger occupancy during peak (A) and non-peak (B) travel times for the 3 types of vehicles.** Abbreviations: CO_2_, carbon dioxide; ppm, parts per million.

Figure 4 shows the correlation between the concentration of carbon dioxide and the percentage of maximum recommended passenger occupancy during peak and non-peak travel times for the 3 types of vehicles. There was a moderate positive correlation between the concentration of carbon dioxide and the percentage of maximum recommended occupancy for the buses during peak (Figure 4A) and non-peak (Figure 4B) travel times. For the shuttle vans, there was a weak positive correlation between the concentration of carbon dioxide and the percentage of maximum recommended occupancy during peak travel times (Figure 4A) and a weak negative correlation during non-peak travel times (Figure 4B). During peak travel times, the percentage of maximum recommended passenger occupancy in the busses never exceeded 65%, whereas the occupancy in the shuttle vans often exceeded 70% and occasionally exceeded 100% recommended occupancy.

### Intervention to Improve Ventilation on the Shuttle Vans

**Figure 5.**
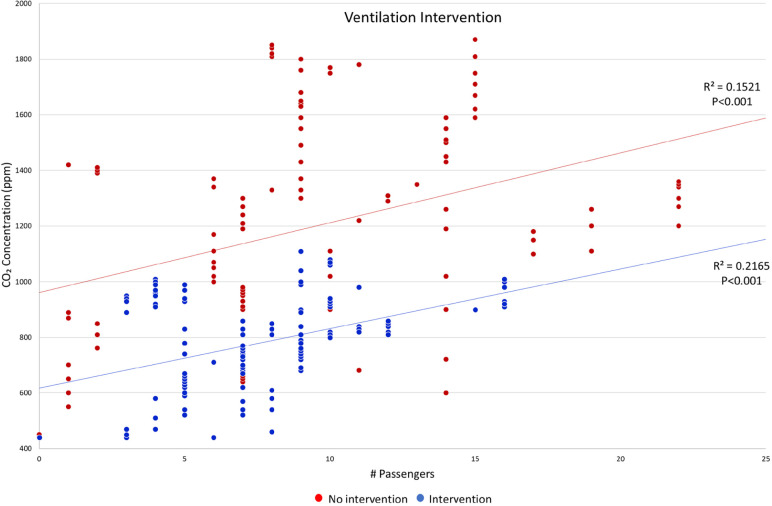
**Concentrations of carbon dioxide during the 4 intervention trips in comparison to the 4 no intervention trips.** Abbreviation: CO_2_, carbon dioxide

During the intervention trips, the drivers of the shuttle vans turned on the existing HVAC system to the medium or high setting during non-peak and peak travel times. Figure 5 shows the concentrations of carbon dioxide during the 4 trips with the intervention in comparison to 4 trips with no intervention. Using a linear regression model, we did not detect any difference in the slopes of the intervention vs no intervention trips (interaction *P*=0.63), but the concentration of carbon dioxide was significantly lower during the intervention vs no intervention trips (intervention effect *P*<0.001). The mean and peak concentrations of carbon dioxide were 785 and 1,080 ppm, respectively, during the intervention trips vs 1,042 and 1,850 ppm, respectively, during the no intervention trips.

## DISCUSSION

Public transportation provides an essential service, improving social inclusion and access to education and employment opportunities. Given the risk for transmission of respiratory viruses in motor vehicles used for public transportation [7–15], there is a need for efforts to assess and optimize ventilation to reduce the risk for aerosol-mediated transmission. Here, we demonstrated considerable variability in the quality of ventilation in 3 types of vehicles based on carbon dioxide monitoring. In large articulated buses with 2 HVAC systems, carbon dioxide concentrations always remained below 800 ppm, whereas in standard buses, carbon dioxide concentrations rose above 800 ppm during peak travel times and, on some trips, during non-peak travel times. On shuttle vans with the HVAC system off, carbon dioxide rose above 800 ppm on all trips during peak and non-peak travel times. During peak travel times, there was a positive correlation between the percentage occupancy of the vehicles and the carbon dioxide concentration.

Our findings have several implications for efforts to prevent transmission of respiratory viruses on public transportation buses and vans. First, the ventilation system should be on whenever passengers are on board and the windows are closed. In the shuttle vans, the intervention involving operation of the ventilation system resulted in a significant reduction in carbon dioxide concentrations. Operation of the ventilation system may be particularly important when vehicles are parked and during city driving [7, 8]. Second, passengers wishing to reduce their risk might consider avoiding peak travel times when ventilation may not be adequate for the number of vehicle occupants. They should also be aware that ventilation may vary considerably among different types of vehicles. Finally, operators of transportation systems should be aware that exceeding maximum recommended occupancy may increase risks to passengers and drivers.

Our results highlight the potential for use of carbon dioxide monitoring to assess and optimize ventilation. Such measurements could be a useful means for officials from public transportation companies and schools to assess the adequacy of ventilation on their buses and vans. Querol et al [21] recently reported that public transport buses in Barcelona, Spain, with increased air circulation had decreased carbon dioxide levels, and adequate air circulation could be achieved through operation of ventilation systems and opening windows. If carbon dioxide monitoring is used, it is important that the limitations of the technology are understood [1, 3, 16]. Carbon dioxide monitoring does not account for filtering of recirculated air, which may reduce risk for aerosol transmission [1, 16]. Exposure to increased carbon dioxide levels has been linked to acquisition of tuberculosis [22]. However, exposure to elevated carbon dioxide levels has not been associated with increased risk for COVID-19.

Our study has some limitations. We conducted testing in only 7 transportation routes with 3 types of vehicles. Additional studies are needed with other vehicle types. We did not gather information on the ventilation system settings in the vehicles. Setting the ventilation system at maximal levels may increase efficacy. We did not examine the potential for fans to increase transmission risk if they direct airflow from an infected source patient to susceptible individuals. Previous studies have suggested that patterns of airflow might have contributed to long-distance transmission of large and small droplets containing SARS-CoV-2 in settings such as restaurants, patient transport vans, and double-occupancy patient rooms [13, 23, 24]. Our investigation did not address measures other than ventilation that might affect transmission risk in vehicles (eg, use of facemasks and physical distancing). Finally, in the standard and articulated buses, the percentage of maximum recommended occupancy never exceeded 65%. Therefore, we cannot exclude the possibility that ventilation on the articulated buses could be inadequate in the setting of 100% or greater occupancy.

In conclusion, we demonstrated substantial variability in the adequacy of ventilation in public transportation buses and university shuttle vans. A simple intervention involving operation of the existing ventilation system on the shuttle vans was effective in improving ventilation. Our findings suggest that there is a need for efforts to assess and optimize ventilation to reduce the risk for aerosol-mediated transmission on public transportation buses and vans. Carbon dioxide monitoring may provide a useful tool to assess and improve ventilation.
